# Association between 24-hour urine sodium and potassium excretion and diet quality in six-year-old children: a cross sectional study

**DOI:** 10.1186/1475-2891-11-94

**Published:** 2012-11-15

**Authors:** Oddny K Kristbjornsdottir, Thorhallur I Halldorsson, Inga Thorsdottir, Ingibjorg Gunnarsdottir

**Affiliations:** 1 Unit for Nutrition Research, Landspitali-University Hospital, Eiriksgata 29, Reykjavik 101, Iceland; 2 Faculty of Food Science and Nutrition, School of Health Sciences, University of Iceland, Eiriksgata 29, Reykjavik 101, Iceland

**Keywords:** Sodium, Potassium, Children, 24h urinary excretion, Diet quality

## Abstract

**Background:**

Limited data is available on sodium (Na) and potassium (K) intake in young children estimated by 24 hour (24h) excretion in urine. The aim was to assess 24h urinary excretion of Na and K in six-year-old children and its relationship with diet quality.

**Methods:**

The study population was a subsample of a national dietary survey, including six-year-old children living in the greater Reykjavik area (n=76). Three day weighed food records were used to estimate diet quality. Diet quality was defined as adherence to the Icelandic food based dietary guidelines. Na and K excretion was analyzed from 24h urine collections. PABA check was used to validate completeness of urine collections. The associations between Na and K excretion and diet quality were estimated by linear regression, adjusting for gender and energy intake.

**Results:**

Valid urine collections and diet registrations were provided by 58 children. Na and K excretion was, mean (SD), 1.64 (0.54) g Na/24h (approx. 4.1 g salt/24h) and 1.22 (0.43) g K/24h. In covariate adjusted models Na excretion decreased by 0.16 g Na/24h (95% CI: 0.31, 0.06) per 1-unit increase in diet quality score (score range: 1–4) while K excretion was increased by 0.18 g K/24h (95% CI: 0.06, 0.29).

**Conclusions:**

Na intake, estimated by 24h urinary excretion was on average higher than recommended. Increased diet quality was associated with lower Na excretion and higher K excretion in six-year-old children.

## Background

The best method of estimating sodium (Na) and potassium (K) intake is by analyzing 24-hour (24h) Na and K excretion in urine [1,2], as the use of dietary surveys and food composition databases for estimating Na and K intake may introduce either an over- or underestimation of the actual intake. Studies including 24h urine collections for estimation of Na and K intake in children are relatively few.

Food based dietary guidelines have been established as a result of studies showing that the overall diet quality rather than specific nutrients protects against chronic diseases in adults [3-5]. The main dietary sources of K contribute to a healthy diet and are in line with food based dietary guidelines [6-8]. On the other hand, the main dietary sources of Na in children are considered to be less healthy, including processed meat and fast food dishes [8,9]. Tracking of dietary habits from early childhood into adulthood has shown that children with extremely high levels of Na intake tend to maintain those levels over time [10-12]. Therefore, diet in childhood can be a significant determinant of adult dietary habits even after several decades [13].

The aim of the present study was to gather information about Na and K intake in six-year-old children by 24h urinary excretion. The aim was also to assess the relationship between Na and K excretion and diet quality.

## Methods

### Subjects

The source population were subjects invited to participate in a longitudinal study on nutrition and health of Icelandic six-year-olds who had previously participated in studies on nutrition and health during infancy [14] or at two years of age [15]. Originally, families of 180 infants from four maternity wards around Iceland were invited to participate in the infant study and 130 two-year-old children were randomly selected by the Icelandic National Registry. In the infant study 138 agreed to participate, 27 were lost in follow-up at 12 months of age leaving 111 eligible subjects for the follow up at six years. From the study on two year olds 69 were eligible for the follow-up study at the age of six years, altogether 180 subjects. Each family was contacted by telephone and invited to take part in the study. If consent was obtained, an introductory letter explaining the details of the study was sent by mail. The study was approved by the Local Ethical Committee at Landspitali-University Hospital in Iceland, The National Bioethics Committee and by Icelandic Data Protection Commission. The participation rate in the follow-up study was 73% where 131 completed three day food records [16]. Only children who were living in the greater Reykjavík area were invited to provide 24h urine collections (n=111) due to practical reasons (i.e. closeness to the study centre), of which 79 agreed. Three of the children providing 24h urine collections returned incomplete food records, resulting in 76 subjects eligible for the present analysis.

### Weighed food records

Parents kept weighed food records for their children for three consecutive days including one weekend day and 2 week days using a kitchen scale (PHILIPS HR 2385, Austria) around the time of the child’s sixth birthday. Each family received a booklet with which to record all food eaten during this time period. Parents were instructed on how to use the scales and to record the date and time of the meals, specifically to record the brand name or type of food, to include recipes of homemade dishes, and record all drinks and vitamin intake. The data was entered into an interview-based nutrient calculating program, ICEFOOD, designed for the national dietary survey of The Icelandic Nutrition Council [17]. Nutrient losses due to food preparation were included in the calculations. This program included 452 food codes or recipes from the Icelandic Nutrition Council, based on 394 food items from the National Nutrition Database, ISGEM.

### Na and K excretion

Parents and caretakers were given both verbal and written instructions in assisting children to collect a 24h urine sample on one of the three days of food recording. Each child was provided with a urine collection bottle, a backpack in which to carry the jug and three 80 mg PABA tablets (PABA check, The royal veterinary and agricultural pharmacy, Cophenhagen). On the first morning of the urine collections, instructions were given to discard the first specimen, and from then on to collect all specimens for up to 24h, up to and including the first specimen the following day. Subjects were asked to take three 80 mg PABA tablets, one tablet during each main meal on the same day as the urine collections. On return to the laboratory, urine volume was recorded. PABA check [18] was used to validate completeness of urine collections. Collections that contained 85% or more of the PABA ingested were considered complete [19]. Recovery between 50% and 85% was adjusted according to a formula developed by Johansson and Bingham 1999 [20]: Na excretion = excretion [mg/day] + (0.82 * (93-PABA recov) and K excretion = excretion [mg/day] + (0.60 * (93-PABA recov)).

Na and K concentration was measured immediately by flame emission photometry [21] at Landspitali University Hospital. The remainder of the samples were stored at −20°C for later analysis of PABA which was measured colorimetrically at Forskningsinstitut for Human Ernæring in Cophenhagen, Denmark [22].

### Diet quality

Adherence to the Icelandic food based dietary guidelines (FBDG) was used to assess diet quality score. Portion sizes used to determine diet quality score were adjusted to reflect the 20% lower energy needs of six-year-olds compared to an adult [23]. The FBDG are based on six recommendations.: ≥400 g fruits and vegetables, ≥34 g fish, ≥5 g fish liver oil and ≥400 g milk and milk products (or 200 g milk and milk products and 20 g cheese). The Public Health Institute of Iceland [6] recommends K intake ≥2 g/d for children 6–9 year old and the Nordic Nutrition Recommendations [24] ≤0.5 g salt/1000 kJ (0.5 g salt/239 kcal) for children 2–18 years old, corresponding to about 3.2 g salt daily (according to energy intake of 1530 kcal/d in the present study). Fiber consumption of at least 11 g/day was used in the evaluation of diet quality score as an indicator of whole grain cereals [25]. One point was obtained by following each guideline, for a maximum of six points. Diet quality score was divided into four groups based on adherence to FBDG, those following one, two, three or at least four of the dietary guidelines.

### Anthropometrics

Height and weight of study participants were measured at Landspitali – Children’s Hospital. Subjects wore light weight clothing and no shoes. Height was measured to the nearest 0.1 cm using a ulmer stadiometer, Busse design (Nersinger Straβe 18, 89275 Elchingen, Germany), and weight was measured to the nearest 0.05 kg using a Taniter BWB-620 electronic scale (2625 South Clearbrook Drive, Arlington Height, Illinois 60005, USA).

### Statistical analysis

Statistical analyses were conducted using SPSS for Windows, version 17 (SPSS Inc, Chicago). Descriptive analyses (mean and standard deviation) were used to describe the characteristics of study participants. A visual inspection of histograms suggested that Na and K urinary excretion was normally distributed. Independent samples *t*-test was used to test the difference between boys and girls and to determine whether Na and K excretion was significantly different between those who follow each food based dietary guideline and those who do not.

To examine the association between diet quality score and Na and K excretion we used multivariate linear regression analyses where gender and energy intake were included as covariates. We included gender as a covariate to account for potential sex dependent differences in behavioral and physiological factors. Total energy intake was included as those with high intake were more likely to meet the food based dietary recommendations (as cutoffs in grams/day were used), while simultaneously having higher intakes of Na and K.

## Results

Of 76 children returning the 24h urine collection and also had complete dietary data, 18 were excluded due to incomplete urine collections according to PABA recovery. More than 85% of the PABA was retrieved in the urine collections of 28 subjects, while excretion from 48 subjects was adjusted (PABA recovery between 50% and 85%). Characteristics of the subjects (n=58), and information on Na and K excretion is shown in Table 1. The average Na excretion was 1.66 g/24h, corresponding to 4.16 g NaCl (table salt). Less than one third (29%) had Na excretion corresponding to an intake below the recommended salt intake of 3.20 g/d [24]. The average K excretion was 1.21 g/24h. No significant gender difference was observed. Based on the three-day food records, mean Na intake was 1.94 g/day (4.86 g table salt/day) and mean K intake was 1.91 g/day. Cereals were found to provide 43% of the total Na in the diet, spices 17%, dairy products provided 15%, meat 11% and 14% came from other sources. Dairy products (32%), fruits and vegetables (22%) and cereals (15%) where found to be the main dietary sources of K.

**Table 1:** Characteristics of study participants

	**N=58 (52% boys)**
Age, months (sd)	72 (1.0)
Height, cm (sd)	119 (4.6)
Weight, kg (sd)	23 (3.1)
Systolic blood pressure, mmHg (sd)	110 (11.1)
Diastolic blood pressure, mmHg (sd)	64 (11)
Urine volume, mL/24h (sd)	648 (284)
***Na excretion***	
mmol/24h (sd)	71 (23)
mmol/L (sd)	120 (44)
g/24h^1^ (sd)	1.64 (0.54)
g/kg/24h (sd)	0.07 (0.02)
***K excretion***	
mmol/24h (sd)	31 (11)
mmol/L (sd)	52 (20)
g/24h^2^ (sd)	1.22 (0.43)
g/kg/24h	0.05 (0.02)
Na/K excretion	1.6 (1.3)

^1^Na excretion corrected for PABA = excretion in mg/day + (0.82*(93-PABA recov)) [20].

^2^K excretion corrected for PABA = excretion in mg/day + (0.60*(93-PABA recov)) [20].

Table 2 shows the proportion of children meeting each of the guidelines used to estimate the diet quality index. Greatest adherence was found for dairy products and fish where 61% and 41% of the children, respectively, had consumption in line with the recommendations. Children who consumed dairy products and dietary fiber in line with the recommendations had significantly greater K excretion than those who did not meet the recommendations (p=0.01) and (p=0.02), respectively.

**Table 2:** **Na and K excretion (g/24h) according to adherence to food based dietary guidelines**[6]

	**Recommendation**	**Children follow (n=58 [n (%)]**	**Na excretion (Mean (sd))**		**K excretion (Mean (sd))**	
			**Following FBDG**	**Not following FBDG**	**Following FBDG**	**Not following FBDG**
Fruits and vegetables	≥400 g/d^1^	5 (8.5)	1.89 (0.99)	1.62 (0.48)	1.44 (0.33)	1.19 (0.43)
Fish	≥34 g/d^1^	24 (40.7)	1.60 (0.51)	1.68 (0.56)	1.34 (0.52)	1.12 (0.34)
Fish liver oil	≥5 g/d^1^	9 (15.3)	1.84 (0.62)	1.61 (0.52)	1.12 (0.38)	1.23 (0.44)
Dairy products	≥two servings/d^1^	36 (61.0)	1.65 (0.62)	1.65 (0.39)	1.32 (0.46)	1.04 (0.32)^4^
Fibre	≥11 g/d^2^	22 (37.3)	1.59 (0.49)	1.67 (0.56)	1.38 (0.48)	1.12 (0.36)^4^
Salt	≤3.2 g/d^3^	17 (28.8)	1.11 (0.18)	1.88 (0.48)^5^	1.33 (0.48)	1.16 (0.40)

*sd*: Standard Deviation.

^1^[6] adjusted according to [23].

^2^[25].

^3^[24].

^4^p<0.05 between following FBDG and not following FBDG.

^5^p<0.01 between following and not following FBDG.

The average Na and K excretion according to diet quality score is shown in Table 3. In covariate adjusted models Na excretion decreased by 0.16 g Na/24h (95% CI: 0.31; 0.06) per 1-unit increase in diet quality score (score range: 1–4) while K excretion was increased by 0.18 g K/24h (95% CI: 0.06; 0.29). Excluding the salt recommendation from the definition of diet quality did not change the findings.

**Table 3:** Mean (sd) excretion of Na and K (g/24h) in urine and Na/K ratio according to diet quality and the association between diet quality and excretion

	**Diet quality score**^**1**^							
	**1**	**2**	**3**	**4**	**Unadjusted**	**p**	**Adjusted**^**2**^	**p**
	**(n=13)**	**(n=24)**	**(n=10)**	**(n=11)**	**B (95% CI)**		**B (95% CI)**	
Na (sodium)	1.70 (0.35)	1.75 (0.73)	1.45 (0.27)	1.45 (0.87)	−0.10 (−0.25; 0.04)	0.06	−0.16 (−0.31; -0.06)	0.03
K (potassium)	1.09 (0.29)	1.08 (0.39)	1.51 (0.55)	1.59 (0.29)	0.19 (0.08; 0.29)	<0.01	0.18 (0.06; 0.29)	<0.01
Na/K ratio	1.7 (0.6)	2.1 (2.1)	1.1 (0.4)	1.0 (0.7)	−0.25 (−0.60; 0.10)	0.07	−0.28 (−0.66; 0.11)	0.06

^1^Follows one of the FBDG; 2: Follows two of the FBDG; 3: Follow three of the FBDG; 4: Follows at least four of the FBDG. [6,23-25].

^2^Adjusted for gender and energy intake.

## Discussions

In the present study Na and K excretion were associated with diet quality among six-year-old children. Na excretion in this study of six-year-old children was 1.66 g/24h (0.07 g/kg/24h), that corresponds to about 4.2 grams table salt. The average consumption of salt worldwide is generally high, particularly in industrialized countries, and the results from the present study are in line with previous findings [26-28]. In the Nordic nutrition recommendations from 2004 [24] ≤0.5 g salt is recommended per 1000 kJ (0.5 g salt/239 kcal) for children 2–18 years old. The average energy intake in the present study was 1530 kcal/day, so the average salt intake should have been close to or below 3.2 g salt daily. Less than one third (29%) of the children in the present study had Na intake in line with the recommendation [24]. Na is part of various additives and hence added to most foods, either by the industry or in cooking. The high Na intake observed in this population is of concern and should be recognized by health authorities. In the population studied lower content of salt in cereals (including bread) could significantly contribute to lower Na intake as this food group provided 43% of the total Na consumed. A reduced salt intake of 42% (IQR: 7%-58%) was found to be associated with 1.17 mmHg decrease in systolic (95% CI: -1.78 to −0.56; p<0.01) and 1.29 mmHg diastolic (95% CI −1.94 to −0.65, p<0.01) blood pressure in a meta analysis including children with mean age of 13 years [29]. From a population viewpoint, a reduction in BP of 1.1 mmHg in this age group would have major effects of preventing cardiovascular disease in the future [29].

K excretion in the present study was 1.21 g/24h or 0.05 g/kg/24h. Few studies exist on K excretion but two studies examined 8–9 year old children and reported excretion of about 1.80 g/24h [28] and 2.00 g/24h (0.07 g/kg/24h) [30]. Another study on 3–5 year old children showed K excretion of 1.00 g/24h or 0.05 g/kg/24h [27]. It is often challenging to compare values from studies on children, mainly due to the different ages and body weights of the young subjects. To ease the comparison, it might be convenient to use the per kilogram approach. K excretion was associated with many of the components of the diet quality index used in the present study, such as dairy and whole grain (fiber). K excretion appeared to be higher among those children following the recommendations on fruit and vegetable intake, but the number of subjects following the recommendations was too few to draw any meaningful conclusions. This is consistent with previous studies of low fruit and vegetable consumption of Icelandic children, which is lower than in many other European countries [23].

A linear trend was observed between decreased Na and increased K excretion and increased adherence to diet quality in the current study. Only a few studies have assessed the association between diet quality and excretion of Na or K and none of them included children. K excretion was found to be associated with diet quality in a study of adults with kidney stones (r=0.23, p<0.01) [31]. The recommended food score was used as an index of healthy diet, which contained food groups such as vegetables, fruits, whole grains, low fat dairy, fish and poultry, similar to the present study. Adult nephrolithiasis patients from the Health Professionals Follow-up Study and the Nurses' Health Studies (NHS) I and II collected 24h urine samples and semiquantitative food frequency questionnaires. In the Dietary Approaches to Stop Hypertension (DASH) trial, a dietary DASH score was given based on seven components: high intake of fruits, vegetables, nuts and legumes, dairy products, and whole grains and low intake of sweetened beverages and red and processed meats. It was found that higher DASH scores were associated with higher K in all three cohorts (*P* for trend all ≤0.01) [32]. In 12 healthy adults, a two-week long elimination of fruits and vegetables from the diet resulted in a decrease in urinary K of 62% (p<0.05), assessed from 24h urine collection [32].

### Strength and limitations

The strength of the study is an accurate food record for three days and a valid 24h urine sample. The fact that we only had one 24h urine excretion per individual might be considered a limitation as more than one collection is needed for individual assessment of Na and K excretion. However, in the present analysis the Na and K excretion measurements were used on group level according to adherence to food based recommendations. Furthermore, the Na and K intake estimated by the food records are also presented in this report, providing additional information. The urine collections were conducted on one of the three days of food recording and Na and K excretion is known to be an indicator of recent consumption.

## Conclusions

Na intake estimated by 24h urine excretion was, on average, higher than recommended. Increased diet quality was associated with lower Na excretion and higher K excretion in six-year-old children.

## Abbreviations

24h: 24-hour; K: Potassium; Na: Sodium; FBDG: Food based dietary guidelines.

## Competing interests

The authors declare that they have no competing interests.

## Authors’ contributions

IT and IG contributed to design, data collection, interpretation and final writing of the paper. TIH contributed statistical analysis, interpretation and final writing. OKK contributed handling/management, statistical analysis, interpretation and wrote the first draft of the manuscript. All authors read and approved the final manuscript.
